# Knots are not for naught: Design, properties, and topology of hierarchical intertwined microarchitected materials

**DOI:** 10.1126/sciadv.ade6725

**Published:** 2023-03-08

**Authors:** Widianto P. Moestopo, Sammy Shaker, Weiting Deng, Julia R. Greer

**Affiliations:** ^1^ Division of Engineering and Applied Science, California Institute of Technology, Pasadena, CA 91125, USA.; ^2^ Materials Engineering Division, Lawrence Livermore National Laboratory, Livermore, CA 94550, USA.; ^3^ Division of Biology and Biological Engineering, California Institute of Technology, Pasadena, CA 91125, USA.; ^4^ Kavli Nanoscience Institute, California Institute of Technology, Pasadena, CA 91125, USA.

## Abstract

Lightweight and tough engineered materials are often designed with three-dimensional hierarchy and interconnected structural members whose junctions are detrimental to their performance because they serve as stress concentrations for damage accumulation and lower mechanical resilience. We introduce a previously unexplored class of architected materials, whose components are interwoven and contain no junctions, and incorporate micro-knots as building blocks within these hierarchical networks. Tensile experiments, which show close quantitative agreements with an analytical model for overhand knots, reveal that knot topology allows a new regime of deformation capable of shape retention, leading to a ~92% increase in absorbed energy and an up to ~107% increase in failure strain compared to woven structures, along with an up to ~11% increase in specific energy density compared to topologically similar monolithic lattices. Our exploration unlocks knotting and frictional contact to create highly extensible low-density materials with tunable shape reconfiguration and energy absorption capabilities.

## Teaser

Hierarchical materials with micro-sized knots absorb more energy and elongate farther than non-knotted ones, and retain shape.

## INTRODUCTION

Many naturally formed composites are able to attain unique mechanical properties, such as high strength and fracture toughness, that surpass the performance of individual components by using intricate hierarchical ordering (*1*). Architectural hierarchy has been known to allow the activation of multiscale toughening mechanisms in bone and enhance the structural stability of hexactinellid sponges (*2*–*4*). Attaining full hierarchical ordering of natural materials remains a challenge; progress has been enabled by modern fabrication methods capable of manufacturing synthetic materials with complex prescribed geometries and multiple orders of hierarchy (*5*–*8*). Precisely architected arrangements of constituent materials have led to unique material properties including acoustic and photonic bandgaps (*9*, *10*), tunable thermal response (*11*), and impact resistance (*12*, *13*). Incorporating hierarchy into synthetic architected materials, such as by forming structural elements out of beams at a distinctly smaller length scale, has also enabled other desirable mechanical properties, namely, high energy absorption and deterministic failure behavior (*14*, *15*). As advancements in manufacturing techniques continue to expand the available design space, hierarchical architected materials have mostly drawn from interconnected design principles where structural members are fused together at their junctions (*14*, *16*–*19*), such as beam-based lattices whose members are composed of periodic unit cells (*7*, *15*). Extensive experimental, computational, and analytical studies have been conducted on periodic architected materials (e.g., beam-based, plate-based, and triply periodic minimal surface lattices) and nonperiodic ones (e.g., foams and spinodal architectures), most of which have interconnected designs (*20*–*28*). These studies reveal that the unique mechanical attributes, such as multistable reconfigurability and high stiffness- and strength-to-weight ratios, arise from the combination of stretching, bending, and buckling among the structural members (*29*–*32*), as well as from energy dissipation within the constituent materials (*33*). These types of interconnected architected materials suffer from the development of inevitable stress concentrations at the junctions upon global mechanical loading, which deteriorates their strength and stiffness at greater-than-theoretical rate and accumulates damage (i.e., microcracks and localized deformations).

Interpenetrating lattice designs have recently been explored as alternatives to interconnected design (*34*, *35*), showing the potential to achieve multifunctionality while being composed of mostly two interconnected lattices. Exploiting friction between structural members has also been shown as a method to absorb energy without accumulating substantial damage (*36*–*39*), but most designs lack hierarchy to further augment their properties. A different hierarchical design framework has been introduced, in which multiple interweaving fibers are arranged into effective beams within a microlattice that contains no junctions (*40*). These interwoven lattices outperform classical monolithic, interconnected lattices with equivalent unit cell designs by offering two to three times higher absorbed energy per cycle when normalized to the first cycle, >70% greater deformability upon tension, >50% compressive strain without catastrophic failure, and directional compliance unachievable in their monolithic counterparts. The separation among the fibers within each effective beam opens the possibility to implement new kinematics beyond beam-joint and plate-hinge mechanisms (*30*, *41*, *42*). For example, several lessons can be taken from knots, which can be found in a wide range of length scales: from sailing, climbing, and sutures to the entanglement of DNA, protein, and polymer strands (*43*–*46*). The topology of knots has long been a topic of mathematical interest because it uniquely incorporates geometry and noncommutative algebra (*47*), and researchers have discovered, for example, that even in two similarly configured knots, a slightly different twist can lead to diametrically opposite stabilities (*48*–*50*). Mechanics-based studies on physical tight knots have revealed the importance of accounting for constituent material properties in knot failure predictions (*51*–*53*), and analyses of loose knots show their potential to increase energy dissipation and introduce stable tightening and untying mechanisms through careful selection of knot geometry and constituent materials (*54*–*57*).

**Fig. 1.**
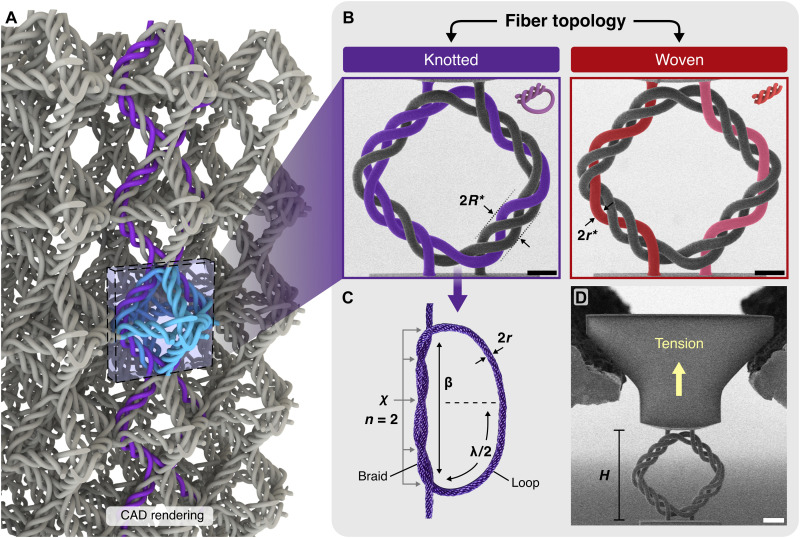
Knotted and woven fiber topologies in hierarchical intertwined materials. (**A**) Computer-aided design (CAD) rendering of a hierarchical octahedron lattice where each unit cell is composed of three rhombuses. The rhombus in the rectangular box is formed by two knotted fibers, one being highlighted in purple and tessellated vertically. (**B**) SEM images with color shading overlaid onto a single fiber in the knotted (left) and woven (right) hierarchical rhombuses, each with two vertically connected fibers. An overhand knot is formed by each of the two fibers (one colored purple and the other uncolored) in the knotted rhombus. Vertical fibers in the woven rhombus are colored red and pink, respectively. (**C**) Photograph of an overhand knot that resembles the purple knot in (A). (**D**) An in situ experimental setup inside an SEM on a representative intertwined rhombus frame. Scale bars, 10 μm (B) and 20 μm (D).

Here, we combine two previously independent concepts, hierarchical architected materials and fiber knotting, to develop building blocks for architected materials with simultaneous high deformability under every loading mode, energy dissipation, fracture resistance, and shape reconfigurability. We classify the fiber topology of hierarchical intertwined materials into two fundamental topologies: knotted and woven (Fig. 1). To elucidate the influence of fiber topology on the mechanical properties of hierarchical intertwined structures, we designed, fabricated, mechanically probed, and analyzed hierarchical woven and knotted rhombus-shaped frames with equal diagonals under quasi-static tension. The probing of a two-dimensional (2D) frame with intertwined beams focuses on an identical substructure inside a 3D unit cell in the lattice, in this case, a rhombus inside an octahedron, while maintaining identical hierarchy of each beam being composed of three intertwined, separate fibers. Our experiments and theory show that the knotted fiber topology enables a new regime of deformation and reconfiguration in architected materials space, i.e., knot tightening. We investigated the effect of interfiber friction on the tying process by (i) applying different surface treatments to the fibers—passivation with a thin (~5-nm-thick) layer of alumina (Al_2_O_3_), systematic irradiation with ultraviolet (UV) light (254-nm wavelength), and aging—and by (ii) conducting quasi-static in situ tensile experiments on all of these samples with different surface treatments. We compare the mechanical response of UV-irradiated versus aged rhombuses, as well as rhombuses with different linear dimensions, to investigate aging mechanisms and size effects in the intertwined polymeric structures.

Intertwined rhombus frames were fabricated out of IP-Dip photoresist using two-photon lithography (Nanoscribe GmbH) with an intended fiber radius r* of 1.69 μm, beam radius R* of 3.5 μm, and rhombus height *H* of 70 μm, as well as rhombuses with twice the linear dimensions (*H* = 140 μm). A 3D octahedron cube unit cell with a relative density (i.e., fill fraction) $\bar{\rho }$ of 5%, calculated as the volume of material in the unit cell compared to the total volume of the unit cell, and width equal to *H* = 70 μm was formed by assembling three rhombuses together, each aligned with one of the three Cartesian principal axes (see Fig. 1A for representation in a lattice). Each beam in the rhombus was composed of three interwoven fibers, and a custom grip was fabricated on top of each rhombus (see Fig. 1D) to enable in situ tensile experiments using a nanoindenter inside a scanning electron microscope (SEM).

## RESULTS

**Fig. 2.**
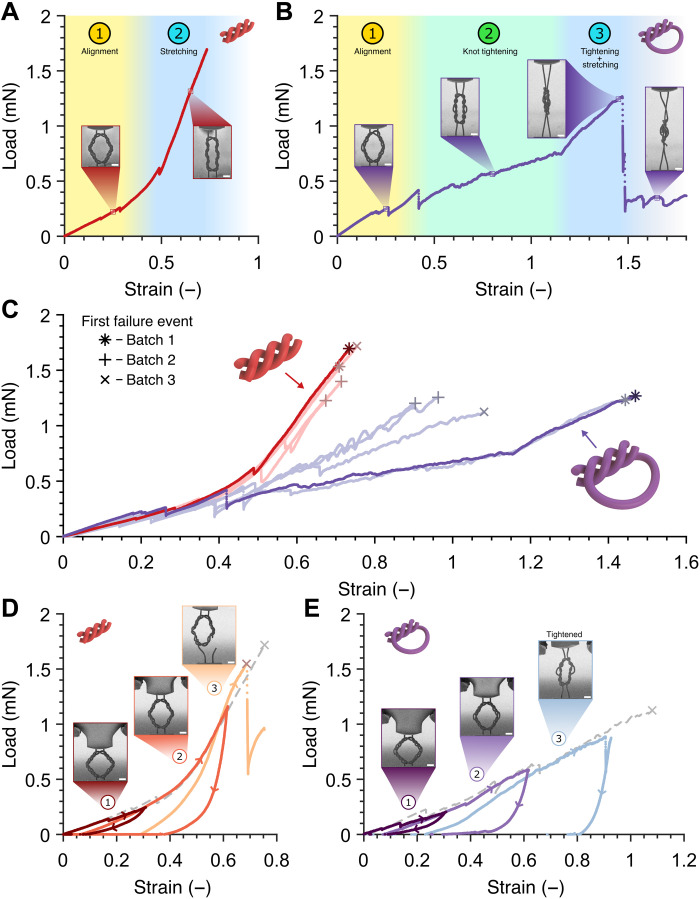
Microscale tensile experiments of hierarchical knotted and woven rhombus frames. (**A** and **B**) Mechanical data and time-lapse images during tension of representative woven (A) and knotted (B) frames pulled up to failure, showing distinct deformation regimes in each fiber topology. Throughout this figure, red data points correspond to woven geometry and purple data points correspond to the knotted one. In (B), mechanical data beyond ~147% strain corresponds to the unraveling of broken fiber(s) after the first failure event. (**C**) Combined tensile response up to the first failure event of five knotted and five woven rhombus frames from three separate rounds of fabrication. Bolder datasets come from the same tests shown in (A) and (B). (**D** and **E**) Load versus strain for woven (D) and knotted (E) rhombuses cyclically loaded in tension, prestrained to incrementally higher extents in each subsequent cycle. The still frames correspond to sample images at the end of each cycle, showing failure in the woven rhombus and stable reconfiguration via knot tightening in the knotted rhombus during the third cycle. Tensile responses up to failure of rhombuses with identical fiber topology originating from the same batch are shown in gray dashed lines. Scale bars, 15 μm.

The influence of fiber topology on the mechanical response of intertwined structures is highlighted in Fig. 2 (A to C), which shows applied uniaxial load *F* versus strain ε and the corresponding time-lapse images during the in situ uniaxial tension experiments on knotted versus woven rhombus frames with a designed height *H* of 70 μm pulled to failure (see also movie S1). Tensile experiments demonstrate distinct regimes of deformation to failure, with the woven topology (Fig. 2A) first undergoing fiber alignment, characterized by a nearly linear region with a slope of ~0.9 mN (regime 1) up to a strain of ~40%, followed by fiber stretching (regime 2) at a five times higher slope in the data up to failure at 73.4% strain. The knotted frame (Fig. 2B) also first underwent fiber alignment (regime 1) with a similar signature up to a strain of ~40%, followed by knot tightening (regime 2) characterized by smoother deformation at a steady stiffness of ~0.5 mN, and the combination of knot tightening and engaged fiber stretching from a strain of ~115% up to failure at 146.9% (regime 3). Figure 2C contains data for five woven and five knotted samples fabricated in three separate batches, and it demonstrates that in the fiber alignment regime, rhombuses of both topologies show similar mechanical signature up to ε ~40%. All woven rhombuses then entered the fiber stretching regime indicated by a five times increase in the load-strain slope, up to incipient failure at the ultimate failure load *F_f_* of 1.22 to 1.72 mN and a corresponding failure strain ε*_f_* of 67.4 to 75.4%. At the transition strain of ε ~40%, the knotted rhombuses were also aligned along the loading direction and continued to deform past the failure strain ε*_f_* of the woven rhombuses via the knot tightening mechanism available to this geometry. Two knotted rhombuses originating from the first batch showed a distinct transition between the knot tightening and fiber stretching regimes at a strain of ε ~115% up to failure at a strain of 144.3 to 146.9% and an applied failure load of 1.23 to 1.27 mN. Knotted rhombuses from different batches contained a less defined tightening-to-stretching transition, failing at a lower strain (90.4 to 96.3% for the second batch and 108.1% for the third) and similar failure loads. The first (ultimate) failure events, depicted with symbols corresponding to the samples’ batch numbers, did not always invoke the breaking of all vertically oriented fibers, with some samples being able to bear load beyond their reported ultimate failure strains (see example in Fig. 2B).

The unique reconfiguration mechanism in hierarchical knotted topology is further showcased in Fig. 2 (D and E), which contains the tensile response of knotted and woven rhombuses from the same batch subjected to several loading/unloading cycles at ~30% strain increments per cycle and under monotonic loading to failure at a strain rate of 1 × 10^−3^ s^−1^ (see also movie S2). These plots indicate that the mechanical response of cyclically and monotonically loaded rhombuses of the same fiber topology matched one another closely at strains beyond the maximum strain of the previous loading cycle. In the first two loading cycles, both the knotted and woven rhombuses elongated via fiber reorientation and uncoiling before returning to their original shapes upon load removal, with slight twisting of the rhombus and a concomitant viscoelastic response present in the unloading region in both topologies. In the third cycle, the woven rhombus failed via fiber rupture around the same failure strain ε*_f_* and failure load *F_f_* as monotonically loaded samples. The knotted rhombus retained its knotted shape following unloading from ε ~90% in the third cycle without any evidence of failure.

**Fig. 3.**
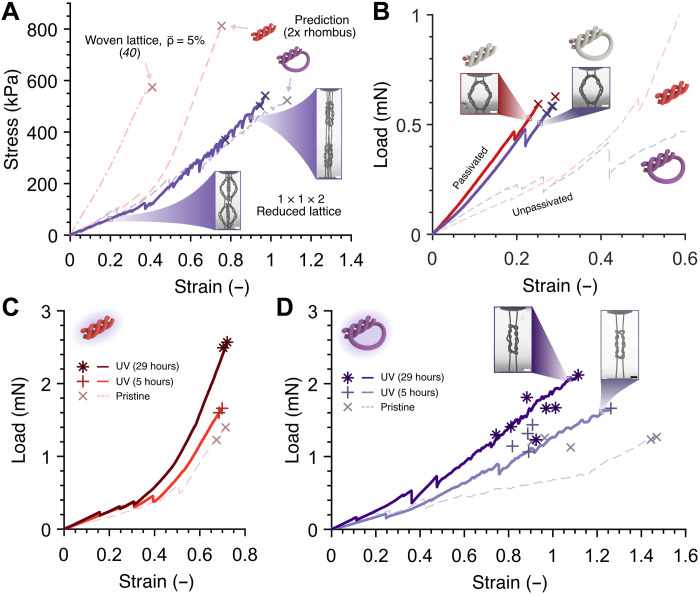
Tensile response of a knotted lattice and effects of passivation and UV irradiation on the mechanical behavior of intertwined architectures. (**A**) Representative tensile response of a tessellation of RUCs, where two knotted rhombus frames are assembled in each unit cell with their knot tightening direction aligned in the loading direction. The first failure event for each experiment is marked with “x.” Predicted responses for a lattice consisting of woven and knotted frames, as well as a woven lattice with a similar relative density from literature, are shown by dashed curves. (**B**) Representative tensile responses up to first failure and corresponding still frames of knotted and woven rhombuses passivated with 5-nm-thick Al_2_O_3_ film. The response of equivalent as-fabricated (unpassivated) rhombuses from Fig. 2 is also shown. First failure events from all experiments are marked with “x.” (**C** and **D**) Representative tensile responses up to first failure events of UV-irradiated and pristine hierarchical woven (C) and knotted (D) rhombuses with corresponding still frames and their first failure events marked with indicated symbols. All woven samples in (C) and some knotted ones in (D) originated from the same batch (see fig. S3D). Mechanical data for pristine hierarchical rhombuses are also shown in Fig. 2C. Scale bars in all SEM images, 15 μm (A, B, and D).

We assembled two knotted rhombus frames, each with their knot tightening direction aligned along the loading path, into a reduced unit cell (RUC) and tested tessellations of RUCs (also termed as reduced lattice) in the knot tightening direction to highlight how the knot tightening mechanism in each frame is translated to a 3D architecture with multiple unit cells (see Fig. 3A). The representative lattice stress σ versus strain ε response is overlaid on top of the predicted stress σ versus strain ε behavior obtained by doubling the tensile responses of knotted and woven rhombus frames to account for the two knotted frames assembled vertically in each unit cell. Figure 3A shows that the reduced lattice stress versus strain response followed the predicted trajectory for a knotted lattice and that it reached an ultimate tensile strength (UTS or σ_UTS_) of 369 to 541 kPa with a corresponding failure strain of 77.3 to 97.1%, both close to the expected UTS of ~522 kPa and a failure strain of ~108% for a knotted lattice (see fig. S1 for more information on RUC and lattice testing).

To elucidate the effect of interfiber friction on the mechanical behavior of intertwined structures, we first passivated the samples identical to rhombuses in Fig. 2C with a 5-nm-thick alumina film deposited via atomic layer deposition (ALD) at 200°C and compared their *F* versus ε response with that of the as-fabricated rhombuses (Fig. 3B). We observed nearly identical load-strain behavior between the two geometries, with the initial slope a factor of ~2.5 to 2.6 times higher than that of the as-fabricated samples, followed by ultimate failure in regime 1 (fiber alignment) at a strain of ~25 to 30%. Passivated knotted rhombuses failed at ε*_f_* = 27.1 to 28.6% and *F_f_* = 0.55 to 0.58 mN, and woven ones failed at ε*_f_* = 25.1 to 29.2% and *F_f_* = 0.59 to 0.63 mN, which are about two to three times lower than those of unpassivated woven frames. We observed a similar trend in the tensile response of cylindrical pillars made of the same IP-Dip resist with radii of 1.69 μm and heights of 10 μm subjected to different postprocessing procedures: (i) as-fabricated samples (termed pristine) and (ii) samples passivated with a 5-nm-thick ALD alumina immediately after fabrication (fig. S2); tensile experiments on the passivated pillars show a ~144 and ~43% increase in Young’s modulus *E* and yield stress σ*_y_* compared to pristine pillars, respectively, as well as a ~27% decrease in σ_UTS_ and an ~88% drop in strain at UTS (ε_UTS_).

We explored how varying fiber bulk and surface properties affect the mechanical behavior of intertwined structures beyond the fiber alignment regime by pulling to failure knotted and woven rhombuses of *H* = 70 μm that had undergone the following treatments: (i) pristine, (ii) irradiated under UV for 5 hours, and (iii) irradiated under UV for 29 hours (Fig. 3, C and D). These experiments revealed that in the fiber alignment regime, i.e., at strains below 40%, the loads of UV-irradiated (ii and iii) woven frames are up to ~80% higher than those of pristine (i) samples (fig. S3A). Increasing the UV radiation time strengthened the woven frames in the fiber stretching regime, which failed around the same ε*_f_*: 67.4 to 71.4% strain for pristine samples, 68.5 to 69.9% for the 5-hour UV-irradiated samples, and 70.3 to 72.1% for the 29-hour UV-irradiated ones, with corresponding *F_f_* values of 1.23 to 1.40 mN, 1.60 to 1.66 mN, and 2.49 to 2.57 mN, respectively.

No clear trend in the difference between pristine and 5-hour UV-irradiated knotted samples was observed; the loads for 29-hour UV-irradiated knotted samples are up to ~90% higher than those of other knotted rhombuses in all regimes of deformation for a given strain (Fig. 3D and fig. S3, B and C). All knotted frames experienced first failure events at higher strains compared to all woven rhombuses and at similar or lower *F_f_* compared to woven rhombuses that underwent the same postprocessing procedures: ε*_f_* = 90.4 to 146.9% and *F_f_* = 1.12 to 1.27 mN for pristine samples, ε*_f_* = 81.7 to 126.2% and *F_f_* = 1.07 to 1.66 mN for 5-hour UV-irradiated samples, and ε*_f_* = 74.4 to 111.4% corresponding to *F_f_* = 1.23 to 2.12 mN for 29-hour UV-irradiated ones.

We investigated the mechanical and surface properties of (i) pristine, (ii) 5-hour UV-irradiated, and (iii) 29-hour UV-irradiated IP-Dip samples via in situ tensile testing of pillars (fig. S2) and x-ray photoelectron spectroscopy (XPS) characterization (fig. S4; see the Supplementary Materials for details on the XPS characterization). These experiments uncovered that greater irradiation time increases the modulus *E* and the yield strength σ*_y_* with a concomitant reduction in tensile strain ε_UTS_. The UTS σ_UTS_ of pillars irradiated for 5 hours decreased by ~10%, while the σ_UTS_ of 29-hour UV- irradiated pillars on another chip increased by ~5% compared with pristine IP-Dip pillars on their corresponding chips. XPS characterization reveals inconsistency with regard to surface properties of similarly treated polymer samples, with no unambiguous surface transformations, such as chain scission (*58*), occurring on a given sample (see the Supplementary Materials).

**Fig. 4.**
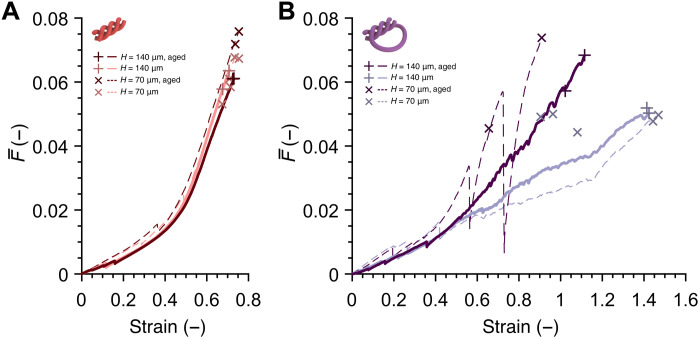
Effects of varying linear dimensions and aging. (**A** and **B**) Representative tensile responses of pristine and aged woven (A) and knotted (B) rhombus frames with a designed rhombus height *H* of 70 and 140 μm. Mechanical data for pristine frames of *H* = 70 μm originated from the data shown in Fig. 2C. First failure events from all samples are marked according to the indicated symbols. The four pristine knotted frames in (B) with ε*_f_* > 140% were fabricated within 2 days of each other on two separate chips and tested within a 24-hour period, while the other knotted rhombuses originated from different batches.

To probe into the influence of feature size on the mechanical behavior of architected materials composed of IP-Dip fibers, which have been reported to lack size effects in modulus and yield strength within 1 to 10 μm (*59*), we fabricated and tested one batch of larger self-similar pristine knotted and woven frames with designs and printing parameters identical to those shown in Fig. 2 and with double the linear dimensions (*H* = 140 μm). We define a normalized load $\bar{F}$ as the applied load *F* divided by the product of the Young’s modulus of pristine IP-Dip *E*_pr_ and the fiber cross-sectional area *A* = πr*^2^. Figure 4 conveys $\bar{F}\;\mathrm{versus}\;\varepsilon$ data for woven and knotted frames and does not show discernible differences between their mechanical responses. These experiments also show that pristine knotted frames attain an average of ~75%, and a maximum of ~107%, increase in ε*_f_* compared to pristine woven samples.

We also performed tensile-to-failure experiments on the knotted and woven frames that had been aged under normal laboratory conditions for more than 75 days and compare their $\bar{F}$ versus ε response in Fig. 4 (A and B). Pristine samples were tested not more than 9 days after fabrication, and the mechanical behavior of pristine versus aged woven frames is virtually identical with the latter failing at an average of ~10% greater $\bar{F}$ compared to pristine woven samples of the same size. Significant discrepancies can be observed in $\bar{F}$ versus ε data for knotted frames, with the aged samples generally being more than 40% stronger than the pristine ones at a given strain past the fiber alignment regime, and the smaller (*H* = 70 μm) knotted frames deforming via a more pronounced stick-and-slip mechanism. The aged knotted frames with *H* = 70 μm experienced first failure events around an ε*_f_* of 65.5 to 90.9% and a normalized ultimate failure load ${\bar{F}}_{f}$ of 0.046 to 0.074, while the larger, aged knotted samples (*H* = 140 μm) had an ε*_f_* of 102.2 to 111.5% and an ${\bar{F}}_{f}$ of 0.057 to 0.068 [see fig. S5 (A and C) for *F* versus ε plots corresponding to Fig. 4 (A and B)]. Tension to failure of all aged passivated (fig. S5, B and D) samples were somewhat more compliant and deformable compared with their pristine counterparts and also experienced first failure events generally within the fiber alignment regime.

**Fig. 5. F5:**
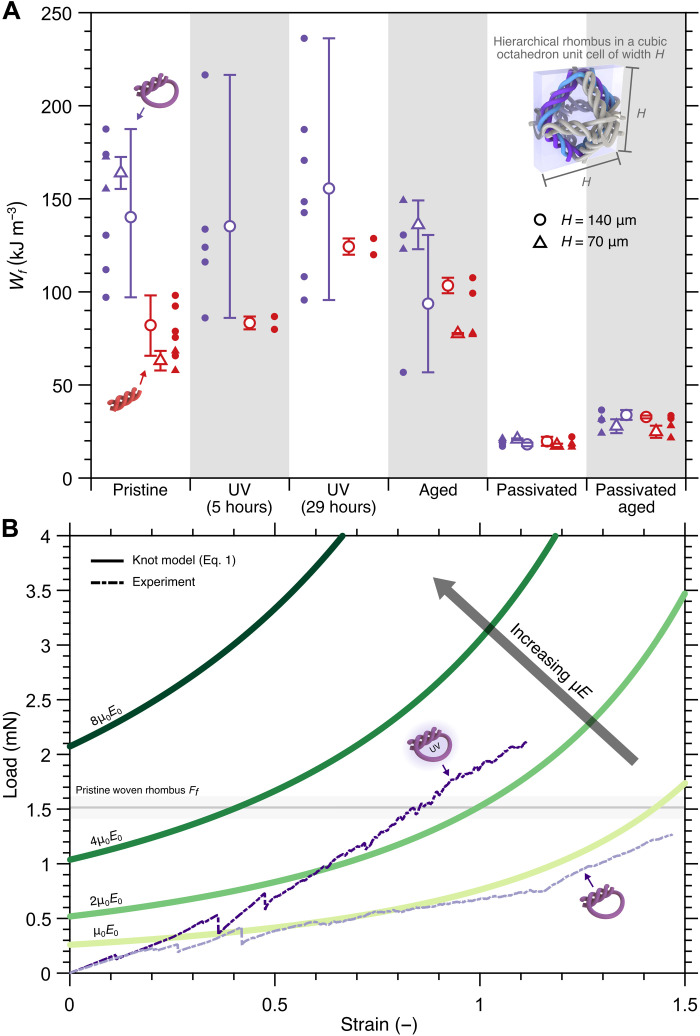
Energy absorption capability and comparison between experiments and an analytical knot model. (**A**) Absorbed energy density up to first (ultimate) failure load, *W_f_*, of all hierarchical intertwined frames tested in this work. The variable *W_f_* represents the contribution of a rhombus frame inscribed in a cubic unit cell with side length *H* to energy absorption. Markers and error bars denote mean and extrema of the datasets, respectively (2 ≤ number of samples ≤ 7). (**B**) Experimental tensile data for pristine and 29-hour UV-irradiated knotted frames of *H* = 70 μm shown alongside analytical predictions with varying fiber properties. The model conveys the tightening of two separate parallel knots with similar geometries and is compared to the experimental results fitted to μ*E* = μ_0_*E*_0_ = 0.2 GPa. The horizontal dark gray line and light gray region represent the mean and SD of the force at first failure events of woven frames (*H* = 70 μm).

To meaningfully compare the energy absorption properties of the intertwined frames with other materials, we introduce the absorbed energy density variable *W_f_* and plot the *W_f_* values for each type of sample tested in this work in Fig. 5A. *W_f_* is defined as the absorbed energy density up to the first (ultimate) failure event and is calculated by taking the area under the *F* versus ε curve up to the strain at first failure and dividing it by *H*^2^, essentially equating *W_f_* as the energy absorption contribution of the frame to a cubic unit cell with side length *H* up to the first failure event. Pristine knotted frames are shown to have 71 to 160% higher average *W_f_* compared to pristine woven samples of the same *H* (Fig. 5A). UV irradiation increases the average *W_f_* of intertwined frames, with knotted samples (*H* = 70 μm) having 11%higher average *W_f_* after 29-hour UV irradiation and woven samples with identical *H* and UV irradiation time having 51% higher average *W_f_* compared to their pristine counterparts. Aged knotted and woven samples of all sizes have 22% lower and 18% higher average *W_f_* than their corresponding pristine samples, respectively. We also observed large drops of average *W_f_* for passivated samples, with passivated pristine knotted samples experiencing the largest drop of ~87% compared to unpassivated ones.

## DISCUSSION

The differences in deformation regimes between knotted and woven frames indicate that fiber topology allows for greater tunability of the mechanical behavior when architected materials are made interwoven without changing the configuration of the higher hierarchical levels, such as the nodal connectivity and unit cell configuration. The ~75% higher average failure strain ε*_f_* in pristine knotted frames compared to woven ones is likely translatable to a lattice framework because the ε*_f_* of woven frames falls within the ε*_f_* of the most extensible IP-Dip hierarchical woven lattices in (*40*). The knotted shape retention upon unloading from the knot tightening regime (Fig. 2E) also presents a novel shape transformation mechanism in an architected material. The ~92% increase in *W_f_* of pristine knotted frames compared to pristine woven frames suggests that friction has a more substantial role in dissipating energy within the knotted geometry than in the woven one because of the addition of the knot tightening deformation regime.

On the basis of the average *W_f_* values reported in Fig. 5A and treating the constriction caused by fibers from other frames in a hypothetical unit cell negligible, each pristine knotted frame in a unit cell is expected to contribute an average *W_f_* of ~147 kJ m^−3^ when pulled in the direction of knot tightening. For an octahedron unit cell with two perpendicular rhombus frames aligned along the pulling axis (Fig. 5A), the unit cell may attain up to *W_f_* ~ 294 kJ m^−3^ in that direction, which is equivalent to a specific energy density *W_s_* up to a first failure load of 4.7 kJ kg^−1^, considering that the unit cell is designed to have a relative density $\bar{\rho }$ of 5% and that the constituent solid (IP-Dip) density is approximately 1250 kg m^−3^ (*59*). This energy absorption is ~34% higher than the average absolute (i.e., up to maximum strain beyond strain at maximum failure load) absorbed energy density, *W*_abs_, of monolithic (non-intertwined) octahedron lattices with a similar relative density and ~105% higher than the average *W*_abs_ of previously reported hierarchical woven lattices (*40*). The reduced lattices in Fig. 3A obtained a specific energy density *W_s_* up to a first failure load of up to 3.9 kJ/kg, which is 11 and 70% higher than the average absolute *W_s_* of previously reported monolithic and woven octahedron lattices, respectively. These substantially higher absorbed energies showcase the ability of knotted lattices to achieve higher toughness compared to topologically similar lattices.

In the case of passivated frames, the similarity in the mechanical behavior of passivated woven and knotted frames, combined with the about two to four times lower ε*_f_* and about two to three times lower *F_f_* compared to their nonpassivated counterparts, suggests that alumina coating diminishes mechanical performance, in contrast to the trend seen in passivated IP-Dip/alumina beam-based microlattices, which become brittle as thicker ALD alumina film is deposited (*60*). The similar mechanical behavior corresponds to nearly identical absorbed energy density *W_f_* between knotted and woven passivated frames, and the lower ε*_f_* and *F_f_* lead to lower *W_f_* between passivated samples and other samples of the same topology and age. The low ε*_f_* and *F_f_* exhibited by the passivated interwoven frames can be explained by the relatively large drop of ~88% in ε*_f_* accompanied by marginal reduction in σ*_f_* during tension of the pristine passivated pillars, likely resulting from the brittle nature of alumina encapsulation (*61*). The ~150 to 160% increase in the initial loads of the pristine passivated frames can also be attributed to the material effect: The higher modulus *E* of 6.25 GPa for passivated pillars results from about two orders of magnitude difference between ALD alumina (~165 GPa) and IP-Dip (~2.56 GPa), as well as the potential effect of thermal curing during the ALD process (*62*). The drop of up to 76% in the failure strain of passivated frames can be explained by the fiber bending-initiated cracks that formed in the ALD alumina film during fiber realignment, which reduced the overall load-bearing capability of the sample through a more uniform load distribution. Greater fiber slenderness ratio, defined as the fiber radius r* divided by the length of the fiber, and a lower initial curvature, as well as tailored surface properties, can be used to induce beyond-alignment deformability in interwoven architectures composed of brittle fibers.

For frames that can deform past the fiber alignment regime, the additional fiber contact and sliding allowed by the knot tightening mechanism in knotted topology grants more control of mechanical behavior via the modification of fiber surface and bulk properties. We apply an analytical knot model for long overhand elastic knots to quantify their deformation and compare it to the load *F* versus strain ε data of knots with varying fiber surface and bulk mechanical properties, as well as knot topology (Fig. 5B). According to the knot model developed by Jawed *et al.* (*54*), the tensile load *F* required to tighten a long overhand elastic knot to a certain end-to-end length *e* = λ + β (analogous to knot size; see schematic in Fig. 1C), where λ is the loop arclength and β is the braid length, can be expressed implicitly as

$$n^{2}\frac{r}{e}=\frac{1}{8\sqrt{3}\pi^{2}}g\left({\left[\frac{384\sqrt{3}\pi }{\mu }\cdot \frac{n^{2}F}{Er^{2}}\right]}^{\frac{1}{3}}\right)$$ (1)

where *n* = (χ − 1)/2 is the unknotting number (i.e., the number of times the fiber must be passed through itself to untie the knot), *r* is the radius of the fiber in the knot, χ is the crossing number (i.e., the number of times the fiber crosses over itself), μ is the dynamic friction coefficient, *E* is the Young’s modulus of the fiber, and *g*(*x*) is a nonlinear function mapping $\frac{\beta }{R}$ to $\frac{\beta^{2}}{eR}$, with *R* being the radius of the knot’s loop. In our adaptation, *r* is set to r*, and the tensile strain ε is calculated by dividing the tensile displacement (*e*_0_ − *e*), where *e*_0_ is the initial end-to-end length, with the initial height *H*_0_. When the model is compared with experimental results, we multiply the tensile load *F* of the model by two to compare it with the tensile load of the frame because there are two intertwined overhand knots in each knotted frame.

The overhand knot model assumes friction to follow Amontons-Coulomb laws of friction (friction force ∝ normal force) and predicts that when two separate, parallel overhand knots with geometries similar to experiments in this work (*n* = 2, *r* = 1.69 μm, and starting *e* = combined rhombus side lengths ≈ 198 μm) are tightened, the difference in magnitude of the applied load *F* for a given strain is roughly proportional to the change in μ*E*. By fitting the parameter μ*E*, the model predicts ananalytical form of *F* versus ε that is similar to experimental data (Fig. 5B). The model also predicts that a ≥4 times increase in μ on an otherwise identical knot may result in a strain at failure of <50% and a lower absorbed energy because the frame would reach the failure load of a woven frame (gray horizontal region in Fig. 5B) at a strain below 50% (see the Supplementary Materials for the discussion on the influence of *n*).

[Fig F5]Close quantitative agreement between the model and experimental data does not immediately validate the use of the overhand knot model for knotted architectures because the model (i) assumes full contact only within the braid of one knot, (ii) does not take into account material nonlinearity and initial curvatures in the undeformed fibers, and (iii) contains restrictions on μ. These limitations, along with a potentially high computational cost for a comprehensive numerical study on knotted architectures, suggest that subsequent studies are necessary to fully untangle the effects of geometrical parameters and material properties on the deformation and energy absorption mechanisms in hierarchical microwoven materials.

The lack of conclusive correlation among the failure strains of frames and pillars subjected to different durations of UV irradiation suggests a transition in rhombus failure initiation mechanism from being primarily influenced by the maximum tensile strain of the constituent material during bending in regime 1, in which a higher pillar ε_UTS_ corresponds to a higher rhombus frame ε*_f_*, to potentially being initiated by more localized deformation when initially curved fibers are straightened in regime 2 while in contact with other fibers. Existing literature on the mechanics of knots have shown that (i) a curvature-based analysis alone is not sufficient to determine the failure properties of physical knots without considering localized deformation when contact occurs and that (ii) variations in the mechanical properties of the constituent material can change the knot failure mechanism for a given number of fiber crossing points (*51*–*53*). Similarities in the mechanical behavior of pristine and 5-hour UV-irradiated knotted rhombuses suggest that the combined effects of surface and bulk mechanical properties of fibers in both types of structures are also similar, which is confirmed through comparisons between pristine and 5-hour UV-irradiated pillars (fig. S2) and surface characterizations (see the Supplementary Materials). Using the overhand knot model as guidance, the higher load levels for 29-hour UV-irradiated knotted rhombuses can be attributed to the 1.3 to 2.1 times higher pillar modulus and post-yield stress compared to pristine and 5-hour UV-irradiated pillars. Polymer chain scission on the surface of 29-hour UV-irradiated fibers may have also increased the dynamic coefficient of friction μ of the fibers, which, in turn, also increases the required load to tighten the knotted rhombuses.

The enhanced contact interactions unique to the knotted geometry offers new ways to induce size effects via size-dependent tribological phenomena, in contrast to previously reported architected materials where smaller-is-stronger size effects have only been shown with a mechanism that relies on using constituent materials with changing bulk mechanical properties in sufficiently small feature sizes (*60*, *63*, *64*). Treating friction between the knotted fibers as coulombic, where the friction force is proportional to the force normal to the friction surface, the knot model effectively predicts that the change in feature size will not affect the size-normalized load $\bar{F}$ as a function of ε, with all other parameters being equal. This prediction is confirmed by the close resemblance between $\bar{F}\;\mathrm{versus}\;\varepsilon$data for pristine knotted rhombuses with *H* = 70 μm and *H* = 140 μm fabricated within 2 days of each other (Fig. 4B and movie S3). Any potential of material size effects between these two rhombus sizes can be ruled out by the similarities in the $\bar{F}$ versus ε data.

Tensile experiments on the aged woven frames also do not show any dependence on rhombus size. Compared to the ~14% increase in $\bar{F}$ at failure between pristine and 5-hour UV-irradiated woven samples from the same batch (Fig. 3C), the increase in $\bar{F}$ at failure between all pristine and aged woven rhombuses is ~27% less, indicating that the bulk tensile properties of aged IP-Dip are closer to the properties of pristine IP-Dip than the UV-irradiated one. For the knotted samples, similarities between $\bar{F}$ versus ε data within the fiber alignment regime of equivalently sized pristine and aged rhombuses suggest that the similarities in the mechanical properties of their constituent materials hold. The higher loads in aged knotted frames compared to pristine ones of the same size may have been caused by an increase in surface friction due to environmentally induced changes in surface properties. A substantial increase in friction interaction can lead to a sufficiently large increase in required load for fiber tightening, leading to sticking behaviors and early failures. Full quantification of the effects of environmental parameters on the mechanical behavior of hierarchical intertwined structures will require a suite of systematically controlled aging experiments within the same batch to rule out sample-to-sample variability.

We introduce knots into interwoven microarchitected materials and demonstrate the ability of knotted structural elements to induce a mechanism for smooth and stable structural reconfiguration via knot tightening, which enhances extensibility and energy absorption in these materials. By varying the bulk and surface properties of constituent cross-linked acrylic-based polymers that comprise the intertwined architectures via passivation and UV irradiation, we find that the maximum tensile strain of the constituent material is crucial to the extensibility of intertwined architectures in the early stage of structural deformation and becomes less important in subsequent stages. A close match between experimental results and an analytical model for overhand knots suggests that the model can be used to aid the optimization of the mechanical properties of knotted architectures, although further exploration is necessary to accurately model the effects of material and geometrical properties on the deformation and energy absorption mechanisms in hierarchical intertwined materials. The unique tightening mechanism demonstrated in this work unlocks ways to create shape-reconfigurable, highly extensible, and extremely energy-absorbing bulk, 3D microarchitected materials with mechanical properties that can be tuned not only by their geometries and bulk properties but also by the surface-driven interactions among the structural elements. Beyond enabling the creation of flexible, tough, and lightweight 3D textiles, such capabilities can be advantageous in applications such as flexible electronics, hernia repair, and devices deployed in the bloodstream where repeated large deformations are encountered and traditionally stiff and brittle constituent materials still need to be incorporated.

## MATERIALS AND METHODS

### Fabrication of intertwined structures and pillars

We first fabricated polymeric intertwined and monolithic structures out of IP-Dip photoresist using two-photon lithography via a commercially available Photonic Professional GT system (Nanoscribe GmbH). All structures were additively manufactured on a silanized Si substrate with laser power and scan speed set at 15 mW and 10 mm s^−1^, respectively. Structures originating from the same batch were printed on the same Si substrate within one printing run. An equal hatching (*d_h_*) and slicing (*d_s_*) distance of 0.1 μm was prescribed for each intertwined rhombus structure and monolithic structure (pillar and plate). The base and top cap of each monolithic pillar was printed using *d_h_* = *d_s_* = 0.1 μm, while the base and top cap for each intertwined structure had *d_h_* = *d_s_* = 0.2 μm. IP-Dip plates of dimensions 3.5 μm by 3.5 μm by 0.3 μm (*L* by *W* by *H*) were fabricated with *d_h_* = *d_s_* = 0.1 μm for XPS analysis. All samples were developed in propylene glycol monomethyl ether acetate for ~20 min and subsequently dried via critical point drying in Autosamdri 931 (Tousimis). To fabricate passivated structures, select polymer structures were conformally coated with 5-nm-thickness Al_2_O_3_ using a plasma-enhanced ALD process inside a FlexAL II system (Oxford Instruments). The chamber was held at 200°C, and trimethylaluminum and O_2_ were used as precursors, resulting in a growth rate of 1.2 Å/cycle.

### UV irradiation

We used a Spectro-UV lamp model ENF-240C (120 V, 60 Hz, and 0.2 A) set to the short-wave setting (wavelength, 254 nm; UV-C radiation) at a distance of maximally 5 cm from the top of the sample to perform UV irradiation on our samples. Samples are marked according to the total duration of UV exposure for each sample, which in our case is either 0 (pristine), 5, or 29 hours. Rhombus frames whose tensile responses are shown in Fig. 3C and fig. S3 (A, B, and D) were fabricated in one batch along with a set of pillars. In all our samples, UV irradiation was performed within 8 days from the previous fabrication/postprocessing step, and mechanical testing was performed within 2 days of UV irradiation.

### In situ mechanical experiments

Uniaxial tension experiments were performed using custom-made tension tips attached to a nanoindenter (InSEM, Nanomechanics Inc.) installed in an SEM (FEI Quanta 200F) to enable in situ imaging of the experiments. Stress (σ) is defined as the engineering stress $\sigma =\frac{F}{A}$, where *F* and *A* are the measured load and the initial cross-sectional area of the sample perpendicular to the vertical loading direction, respectively. Strain (ε) is defined as the engineering strain $\varepsilon =\frac{\delta }{H}$, where δ is the sample displacement and *H* is the initial height of the sample. Displacement values for intertwined structures were obtained from the nanoindenter and corrected using a compliance correction method according to Moestopo *et al.* (*40*) while displacement values for pillars tested in tension were obtained via digital image tracking. No significant deviation is observed between the compliance correction method and digital image tracking for rhombus testing (fig. S6). Unless otherwise noted, each experiment was performed within 9 days after fabrication using a strain rate of 1 × 10^−3^ s^−1^. Structural dimensions of each sample were measured inside the SEM before mechanical testing, and measurements of fiber diameters were used to calculate the effective fiber cross-sectional area for each sample. Because the rhombus design is intended to be adaptable to a cubic unit cell design with side length equal to *H*, the absorbed energy density up to first (ultimate) failure load *W_f_* and absolute absorbed energy density *W*_abs_ are calculated by

$$W_{f}=\int_{0}^{\varepsilon_{f}}\frac{F}{H^{2}}d\varepsilon$$ (2)

$$W_{\mathrm{abs}}=\int_{0}^{\varepsilon^{*}}\sigma \;d\varepsilon$$ (3)

where ε*_f_* is the strain at first (ultimate) failure load, σ is the engineering stress on a lattice structure, and ε^*^ is the maximum tensile strain achieved by the structure during testing. The yield strength of an IP-Dip pillar is defined as the 0.2% offset yield strength.

### X-ray photoelectron spectroscopy

XPS data were collected using a Kratos AXIS Ultra spectrometer (Kratos Analytical, Manchester, UK). The instrument was equipped with a hybrid magnetic and electrostatic electron lens system, a delay-line detector, and a monochromatic Al Kα2 x-ray source (1486.7 eV). Data were collected at pressures of <9 × 10^−9^ torr with photoelectrons collected at 90° with respect to the sample surface normal unless otherwise specified. The electron collection lens aperture was set to sample a 700 μm–by–300 μm spot, and the analyzer pass energy was 80 eV for survey spectra and 10 eV for high-resolution spectra. The instrument energy scale and work function were calibrated using clean Au, Ag, and Cu standards. The instrument was operated by Vision Manager software v. 2.2.10 revision 5. The XPS data were analyzed using CasaXPS software (CASA Software Ltd.).

### Aging

Samples marked as aged were stored in normal laboratory conditions for 75+ days before undergoing mechanical testing.

## Supplementary Materials

### This PDF file includes:

Supplementary Text Figs. S1 to S7 Tables S1 to S4 Legends for movies S1 to S3 References
