# Supplementary material for: Knots are not for naught: Design, properties, and topology of hierarchical intertwined microarchitected materials
Source: Sci Adv. 2023 Mar 8;9(10):eade6725. doi: 10.1126/sciadv.ade6725 (PMC9995035; doi:10.1126/sciadv.ade6725)
Supplement: Supplementary file 1 — Supplementary Text Figs. S1 to S7 Tables S1 to S4 Legends for movies S1 to S3 References [file sciadv.ade6725_sm.pdf]

Science Advances
AAAS

Supplementary Materials for

Knots are not for naught: Design, properties, and topology of hierarchical intertwined microarchitected materials

Widianto P. Moestopo *et al.*

Corresponding author: moestopo1@llnl.gov, widi@caltech.edu

Sci. Adv. **9**, eade6725 (2023)
DOI: 10.1126/sciadv.ade6725

The PDF file includes:

Supplementary Text
Figs. S1 to S7
Tables S1 to S4
Legends for movies S1 to S3
References

Other Supplementary Material for this manuscript includes the following:

Movies S1 to S3

Supplementary Text

Tensile Responses of Structures Composed of Multiple Rhombus Frames

Fig. S1A contains SEM images of typical knotted lattices with the inevitable printing-induced defects pointed by the red arrows. We observed similar warping in all 2x2x2 knotted structures, which is caused by the proximity of the printable build area in our two-photon polymerization system (Nanoscribe) to the edges of the 140x140x140 μm^3 lattices, as well as by the detached horizontal rhombuses. Since the activation of the knot tightening mechanism requires fibers to be able to slide past each other, the observed warping may prevent the fibers within the knots to slide smoothly and instead induce a stick–slip behavior and/or suppress fiber motion. If the fibers within a knot are unable to slide as intended, whether by sticking or constriction, the structure would essentially behave as a woven structure when pulled in tension, which is what we observed in the tensile test result of our 2x2x2 knotted structure (Fig. S1A).

To circumvent the issue of warping and fiber sticking, we designed a simpler unit cell with two knotted rhombus frames aligned vertically, termed *Reduced Unit Cell* (RUC), and tested single RUC and multi-RUC structures in tension as shown in Fig. 3A and Fig. S1, B and C. We varied the fiber radius r^* for each structure to mitigate unwanted sticking and constriction, and we estimated the resulting relative density of each structure by comparing the measured fiber radii with r^* = 1.69 μm for a full knotted lattice with three rhombus frames and a relative density $\bar{\rho}$ of 5%, analogous to the one in Fig. S1A. We observed that the knot tightening mechanism was activated for multi-RUC structures (termed *Reduced Lattice*) in Fig. 3A, resulting in a specific energy density W_s of up to 3.9 kJ/kg, which is 11% and 70% higher than the average W_s of monolithic and woven octahedron lattices in Ref. (*40*), respectively.

Validity of Unknotting Number in the Model

The effect of the unknotting number n in the overhand knot model manifests itself in the loop radius R as a function of strain ε: $R \to r$ as $\varepsilon \to$ 150% for n = 2 in the model, and $R \to r$ as $\varepsilon \to$ 94% for n = 3. These strains represent initiation of transition from knot tightening to fiber stretching regime (Fig. 2B); beyond them the model no longer applies. We measured the highest failure strain of pristine and UV-irradiated knotted rhombuses in our experiments to be ~147%, rendering n < 3 to be more appropriate for comparison with experiments.

X-ray photo-electron spectroscopy (XPS) Characterization

XPS performed on as-printed and 29-hr irradiated samples revealed slight differences in peaks assigned to carbon in several oxidation states, as demonstrated in Tables S1 and S2 (see also Fig. S4). However, the C 1s signal as assigned to the as-printed sample was not consistent between different as-printed samples as seen between Tables S1 and S3, indicating a lack of an unambiguous signal of chain scission in the system.

Table 1 shows a C 1s signal composed of five elements yielded from XPS performed on a single site on one IP-Dip sample measured near the time of synthesis. Full width at half maximum (FWHM) of all peaks other than the π-π* shake-up was constrained between 0.8-1.4 eV as

appropriate for a modern instrument. Notably, a stretch at the expected position of a C=O signal was present despite the absence of carbonyls in the known composition of the photoresist. This may be due to the presence of carbonyls in the undeclared 10% of the IP-dip photoresist as per the MSDS [see Refs. (*65*, *66*) for more information on conducting and interpreting XPS results]. We record the ratio of the C-O:C-C/C-H and O-C=O:C-C/C-H intensities (RSF adjusted) from the sample corresponding to Table 1 for further analysis:

C-O:C-C/C-H: 944.3/2348.4 = 0.4017,
O-C=O:C-C/C-H: 1332.1/2348.4 = 0.5662.

XPS performed on a single site on one IP-Dip sample irradiated for 29 hr yielded a C 1s signal composed of five elements assigned in Table 2. FWHM of all peaks other than the π-π* shake-up was constrained between 0.8-1.4 eV as appropriate for a modern instrument. Again, a stretch at the expected position of a C=O signal was present despite the absence of carbonyls in the known composition of the photoresist. With respect to the pre-irradiation sample, we noted that the ratios C-O:C-C/C-H and O-C=O:C-C/C-H with respect to peak intensities (RSF adjusted) decreased after irradiation (see below); however, given the differing results for the pre-treatment and post-treatment conditions in Tables S3 and S4, we are skeptical of the utility or clarity of this result. The ratio of the C-O:C-C/C-H and O-C=O:C-C/C-H intensities (RSF adjusted) corresponding to Table 2 are as follows:

C-O:C-C/C-H: 1402.6/3539.5 = 0.39627,
O-C=O:C-C/C-H: 1927.8/3539.5 = 0.54465.

The computations below, following the attempted method of quantifying the degree of transformation of C-O and O-C=O functional groups in the irradiation process, demonstrate the potentially widely varying value of the ratio of these components between scans of samples produced under identical conditions. Given the inherent difficulty in differentiating between carbon deriving from an adventitious source versus carbon in a known polymer, these values shed doubt on the ability to accurately derive transformation of surface polymer chains from the collected XPS data, especially when compared to data obtained from 29-hr UV-irradiation of the previous sample. The ratio of the C-O:C-C/C-H and O-C=O:C-C/C-H intensities (RSF adjusted) corresponding to Table S3 are as follows:

C-O:C-C/C-H: 2697.8/4483.4 = 0.60173,
O-C=O:C-C/C-H: 2113.3/4483.4 = 0.47136.

The C-O:C-C/C-H and O-C=O:C-C/C-H intensities (RSF adjusted) corresponding to Table S4 reported below demonstrate the potentially widely varying value of the ratios calculated between

pristine samples and the same samples after 29-hr of UV-irradiation, exhibiting the unreliability of the measure as a gauge of chain scission on sample surfaces:

C-O:C-C/C-H: 3300.2/4623.1 = 0.71385,
O-C=O:C-C/C-H: 2392.5/4623.1 = 0.51751.

The Influence of Tensile Failure Strain of the Constituent Material on Intertwined Frames

As highlighted in Fig. 2, A to C, the load-strain behavior of both the knotted and woven frames are nearly identical in the fiber alignment region (Regime 1) before diverging in subsequent regions. While knotted frames in our work generally reached higher tensile failure strains compared to their woven counterparts, passivated frames of both topologies were the only types of samples that consistently failed within Regime 1 at similar failure strains and loads. To understand the influence of the tensile failure strain of the constituent material ($\varepsilon_{\mathrm{UTS}}$) on the extensibility of intertwined frames, we first estimate the minimum $\varepsilon_{\mathrm{UTS}}$ required to allow the structure to deform past the fiber alignment regime, i.e., the maximum tensile strain induced in a fiber when a helical strand of three fibers is straightened.

For a helix with an effective beam radius R^* (see Fig. 1 and Fig. S7) and a fiber radius r^*, the helix can be parametrically defined as $\bar{r} = (r_h \cos t\,, r_h \sin t\,, c\,t)$, where $r_h = R^* - r^*$ is the helix radius, $c = \frac{\lambda}{2\pi}$ is the helix parameter with λ being the pitch of the helix, and $t = 2\pi n$ where n is the number of helix evolutions. The curvature κ of the helix can be calculated as

$$\kappa = \frac{r_h}{{r_h}^2 + c^2}\,, \tag{S1}$$

and the arc length s can be calculated as

$$s = t\,\sqrt{{r_h}^2 + c^2}. \tag{S2}$$

Assuming the helix arc length stays constant and torsion is negligible, the maximum tensile strain $\varepsilon_{t,max}$ of a fiber straightened to a curvature κ_{s} from an initial curvature κ_0 can be calculated as

$$\varepsilon_{t,max} = \frac{r^*(\kappa_0 - \kappa_{\mathrm{s}})}{1 - (r^*\kappa_0)}. \tag{S3}$$

By taking r^* = 1.69 μm, the initial beam radius ${R^*}_0$ = 3.5 μm, and the straightened beam radius ${R^*}_{\mathrm{s}} = \frac{2\mathrm{r}^*}{\sqrt{3}}$ for the 70 μm unit cell design, we obtain a maximum tensile strain of 9.6% within a given fiber in the helix. Among the pillars that we have tested (see Fig. S2), the passivated pillars were the only samples that consistently failed in tension below the 9.6% straightening strain threshold. This result, coupled with the observation that passivated frames were the only samples that could not deform past the fiber alignment regime, suggests that the tensile failure strain of the constituent material is crucial in determining the possibility of failure of an intertwined frame within Regime 1.

For the non-passivated intertwined frames that deformed beyond the fiber alignment regime, we did not observe any correlation between their failure strains and the failure strains of their corresponding pillars despite a nearly 46% reduction in $\varepsilon_{\mathrm{UTS}}$ between pristine pillars and 29-hr

UV-irradiated ones. To better show the discrepancy between our experimental results and what we would expect if $\varepsilon_{\mathrm{UTS}}$ were as crucial in determining frame failure as in Regime 1, we can roughly estimate the expected failure strain of the frame ε_f by calculating the change in the frame effective strain when fibers are aligned in the loading direction ε_a, the maximum tensile strain within a fiber in the junction during alignment ε_j, and the change in frame effective strain when fibers are stretched ε_s. For a rhombus frame with a 45° internal angle and height H, ε_a can be calculated as

$$\varepsilon_a = \frac{H\sqrt{2} - H}{H} = \sqrt{2} - 1 = 41.4\%. \tag{S4}$$

We estimate ε_j by replacing κ_0 in Equation R3 with the tightest curvature within the junction, which is approximately 0.17 μm^{-1}. While this estimate results in ε_j = 27.2%, which is around 14% higher than the tensile failure strain of 29-hr UV-irradiated pillars, it also assumes that the fibers in the strands are all straightened to tightly contact one another, which is not necessarily the case in experiments (see Fig. 3D in main text). Using this value of ε_j, we calculate ε_s and the estimated failure strain of the frame ε_f as

$$\varepsilon_s = \frac{\lambda_s - \lambda_0}{\lambda_0} + \left(\left(\varepsilon_{UTS} - \varepsilon_j \right) \frac{\lambda_s}{\lambda_0} \sqrt{2} \right), \tag{S5}$$

$$\varepsilon_f = \varepsilon_a + \varepsilon_s, \tag{S6}$$

where λ_0 = 28.7 µm is the pitch of the undeformed helical strand, and λ_s = 34.0 µm is the pitch of the straightened helical strand with all fibers touching. For the case of 29-hr UV-irradiated frames, we can consider $\varepsilon_{\mathrm{UTS}} - \varepsilon_j = 0$ since they were able to deform beyond Regime 1. Our prediction of the failure strain of the frame ε_f, which only considers the straightening of fibers with maximum tensile strain equal to $\varepsilon_{\mathrm{UTS}}$, produces rough ε_f estimates of 88.2%, 66.5%, and 59.9% for pristine, 5-hr UV-irradiated, and 29-hr UV-irradiated frames, respectively.

As an alternative to estimate ε_f for a knotted frame, we calculated the minimum achievable knot radius of curvature R_{min} between the braid and the loop for a given fiber $\varepsilon_{\mathrm{UTS}}$, which corresponds to a specific ε_f. We define the maximum bending strain ε_b of the fiber near the entrance of the knot as follows:

$$\varepsilon_b = \frac{r^* \left(\frac{1}{R_{min}} - \frac{1}{R_{start}} \right)}{1 + \left(\frac{r^*}{R_{start}} \right)}, \tag{S7}$$

where R_{start} = 6.05 µm is the initial radius of curvature between the braid and the loop at zero frame strain. By replacing ε_b with $\varepsilon_{\mathrm{UTS}}$ of a pristine, 5-hr UV-irradiated, and 29-hr UV-irradiated fibers, we obtained the corresponding ε_f: 137% for pristine, 117% for 5-hr UV-irradiated, and 102% for 29-hr UV-irradiated frames.

The estimated ~34-47% higher tensile strain at failure for pristine frames compared to 29-hr UV-irradiated frames is contrary to our experimental results, where ε_f for pristine and UV-irradiated woven frames are close to each other, within 67.4-75.4% (see Fig. 3C). For the knotted frames,

despite a trend of increasing ε_f with an increasing $\varepsilon_{\mathrm{UTS}}$ shown by the samples with the highest ε_f for a given UV-irradiation time, most samples still failed within ε_f = 74.3-111.4% regardless of their UV-irradiation times. Existing literature on the mechanics of knots have shown that (i) a curvature-based analysis alone is not sufficient to determine the failure properties of tight physical knots without considering localized deformation when contact occurs, (ii) variations in the mechanical properties of the constituent material can change the knot failure mechanism for a given number of fiber crossing points (*51*-*53*), and that (iii) a higher surface friction will result in a higher tensile load vs. strain curve (*54*). The tensile load vs. strain response of the intertwined frames, informed by the existing literature on overhand knots, serve as the foundation of our prediction of the frame failure mechanism beyond the fiber aligning regime. A further independent study that includes high-fidelity numerical modeling and/or three-dimensional mapping of the fibers during experiments will be needed to predict the frame failure behavior more precisely.

The Degree of Polymer Crosslinking in UV-Irradiated Samples

As a guideline to estimate the degree of polymer crosslinking (DC) in our samples, we refer to the work by Bauer et al. (*59*), which follows a general methodology to determine the DC of a printed structure and analyzes the trends between DC and mechanical properties. While the mechanical characterization of the samples in Ref. (*59*) mainly focuses on properties obtained from compression testing, a short section in Ref. (*59*) reports tensile properties of up to 9 pillars printed with varying printing parameters. Assuming the general trends in Ref. (*59*) hold for our printing parameters, we compare the values of Young's Modulus and ultimate tensile strength (UTS) from our pillar tension tests with those in Ref. (*59*), and we estimate the DC of our pristine, 5-hr UV-irradiated, and 29-hr UV-irradiated samples to be 39-43%, 43-44%, and 44-45%, respectively.

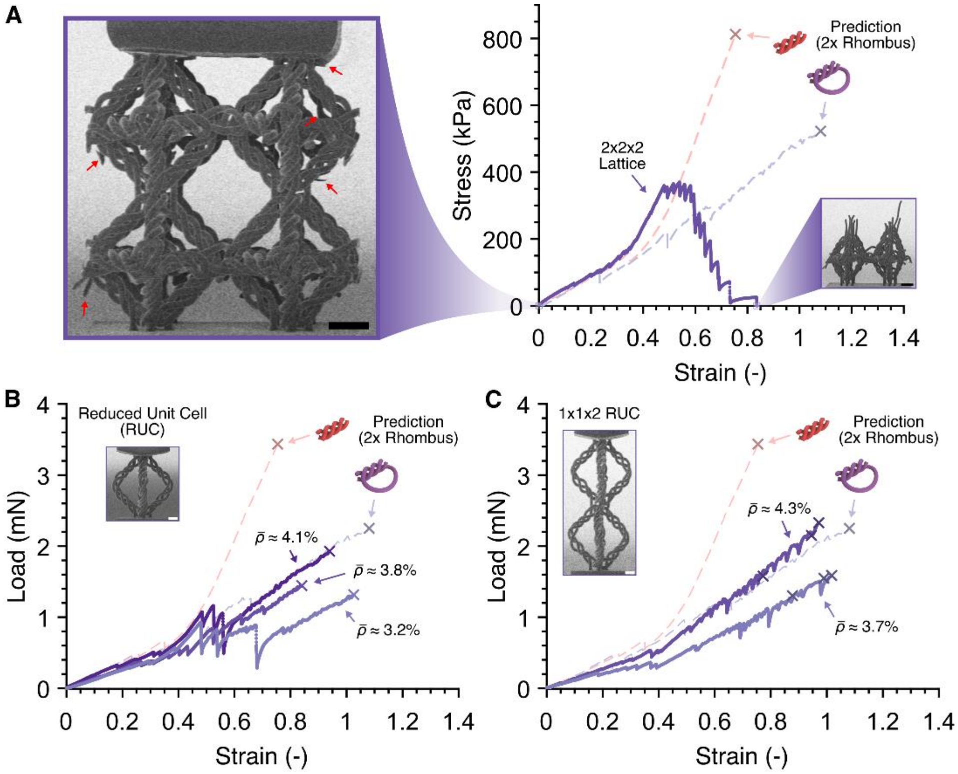

Fig. S1.

Tensile testing of micro-sized structures composed of multiple rhombus frames. (A) Tensile response of a 2x2x2 tessellation of octahedron unit cells with each unit cell consisting of two knotted rhombus frames aligned along the direction of loading and one horizontal woven frame. Red arrows in the inset point to examples of printing defects. Predicted responses for a lattice consisting of woven and knotted frames are shown by dashed curves. **(B** and **C)** Tensile responses of a single (B) and a 1x1x2 tessellation (C) of reduced unit cells, where two knotted rhombus frames are assembled in each unit cell with their knot tightening direction aligned with the loading axis. First failure event from each experiment is marked with “x”, and relative density of each structure is estimated by comparing the measured fiber radii with $r^* = 1.69$ μm for a knotted unit cell with three rhombus frames and a relative density $\bar{\rho}$ of 5%. Predicted responses for woven and knotted lattice frames are shown by dashed curves. Scale bars in all SEM images: 20 μm (A), 10 μm (B and C).

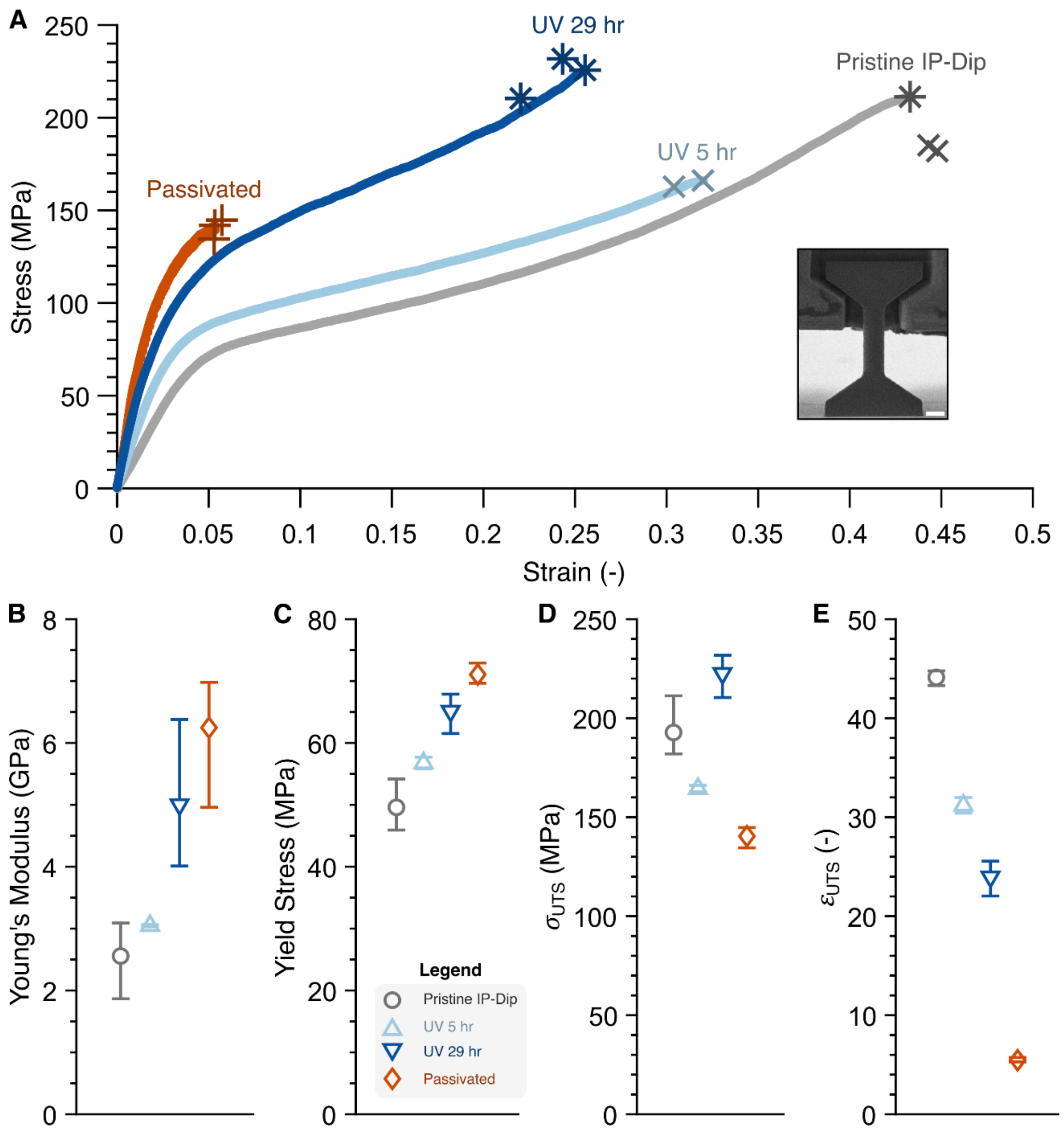

Fig. S2.

Tensile experiments on IP-Dip pillars. (**A**) Mechanical behaviors of IP-Dip pillars that underwent various post-treatment procedures. The pillars had a designed radius of 1.69 µm, identical to the intended fiber radius r^* in hierarchical rhombuses with designed height H of 70 µm. The ultimate failure events corresponding to pillars fabricated in the same batch are marked with the same symbol. Inset shows in situ tensile testing set-up inside a scanning electron microscope (SEM). (**B** to **E**) Young's Modulus (B), yield stress (C), ultimate tensile strength (D), and strain at ultimate tensile strength (E) of IP-Dip pillars shown in (A). Markers and error bars represent mean and extrema of the data sets, respectively. Scale bar: 3 µm.

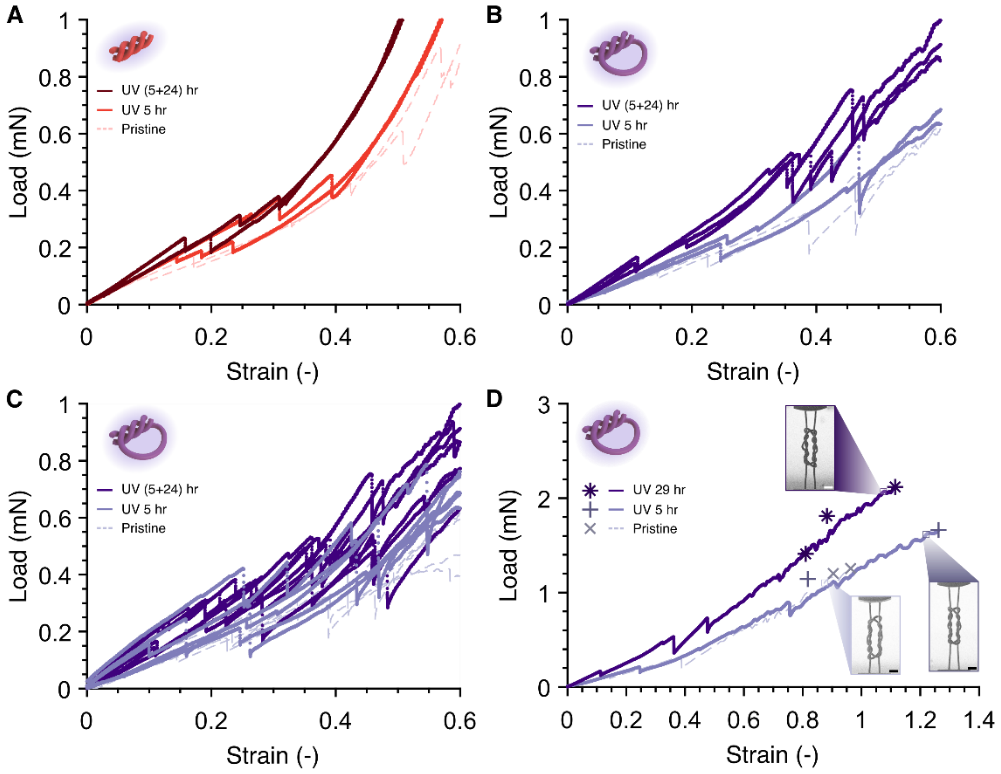

Fig. S3.

Effects of UV-irradiation on the fiber alignment regime of hierarchical knotted and woven frames. (**A** to **C**) Tensile responses within the fiber alignment regime of UV-irradiated and pristine hierarchical woven (A) and knotted (B) frames originating from the same batch. Tensile responses of additional knotted frames from a different batch are shown in (C) alongside those from (B). (**D**) Representative tensile responses up to first failure events of UV-irradiated and pristine hierarchical knotted rhombuses from (B) with corresponding still frames. All first failure events are marked with indicated symbols. Scale bars: 15 μm (D).

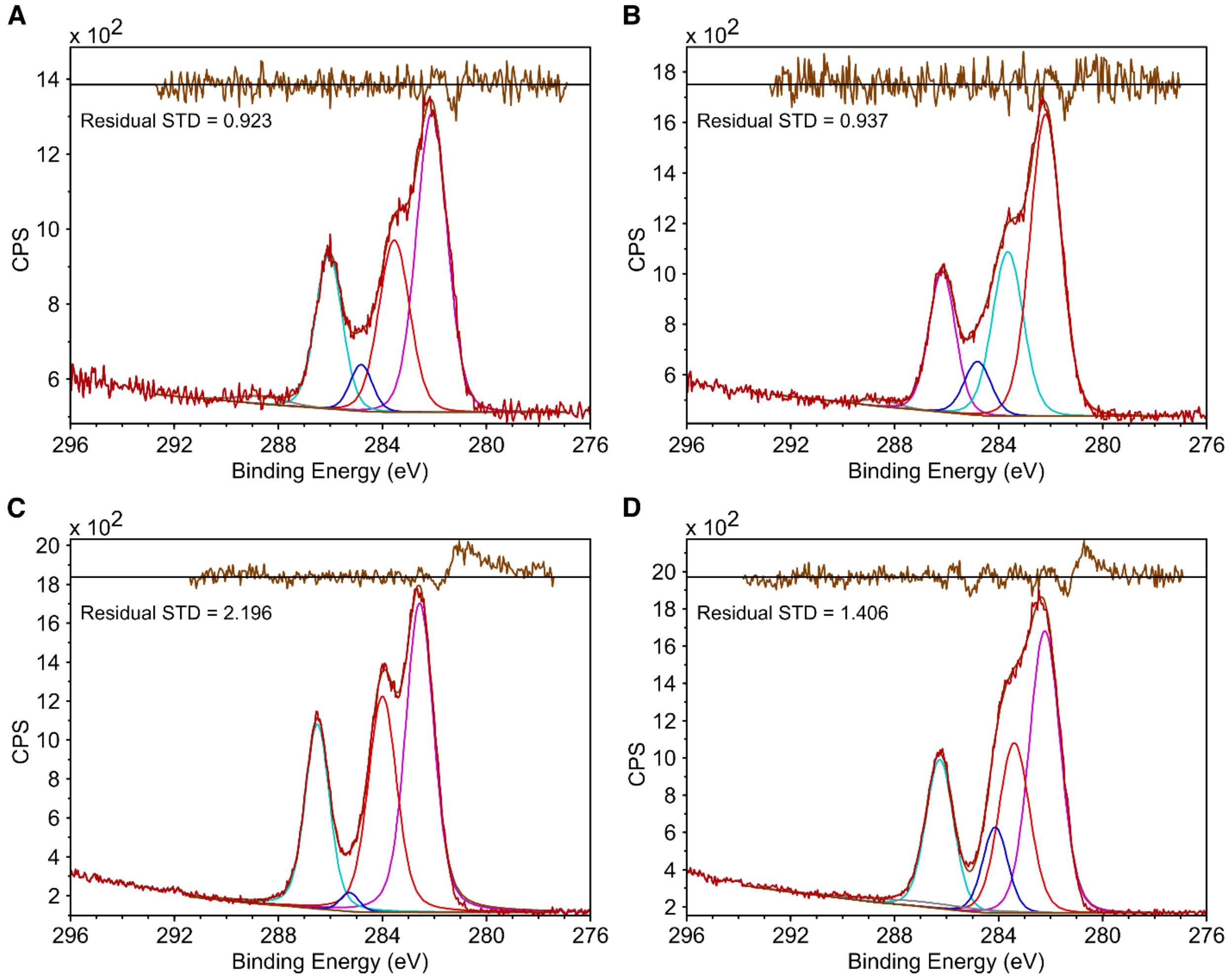

Fig. S4.

XPS characterization of IP-Dip plates. (**A** and **B**) C 1s spectrum of a set of IP-Dip plates without UV-irradiation (A) and after 29-hr UV-irradiation (B). (**C** and **D**) C 1s spectrum of a second set of IP-Dip plates without UV-irradiation (C) and after 29-hr UV-irradiation (D). CPS is photoelectron counts in counts per second.

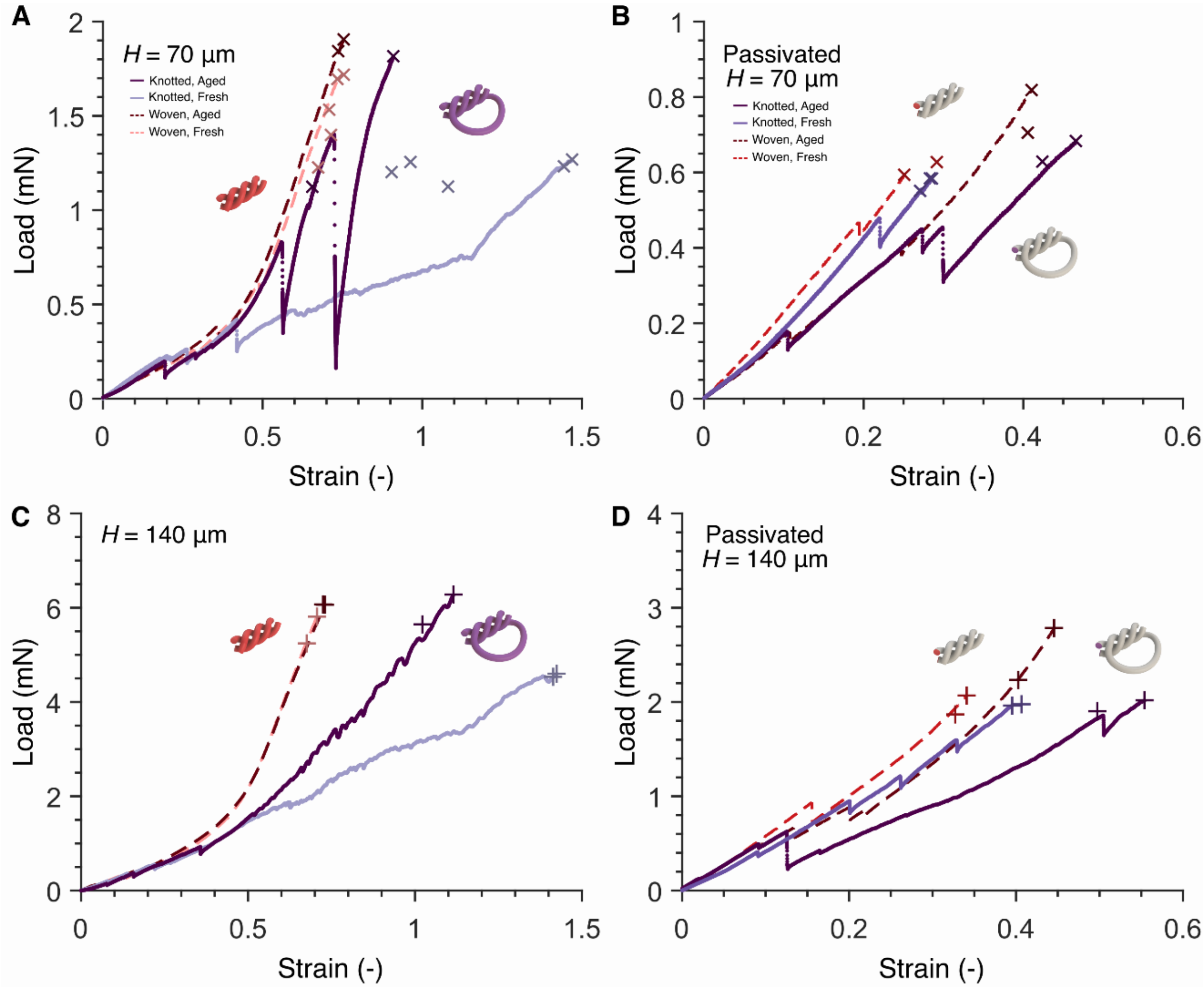

Fig. S5.

Effects of aging on unpassivated and passivated intertwined architectures of varying sizes. (**A** and **B**) Representative tensile responses up to first failure events of pristine and aged unpassivated (A) and passivated (B) rhombus frames of $H = 70$ µm. First failure events are marked by "x". (**C** and **D**) Representative tensile responses up to first failure events of pristine and aged unpassivated (C) and passivated (D) rhombus frames of $H = 140$ µm. First failure events are marked by "+".

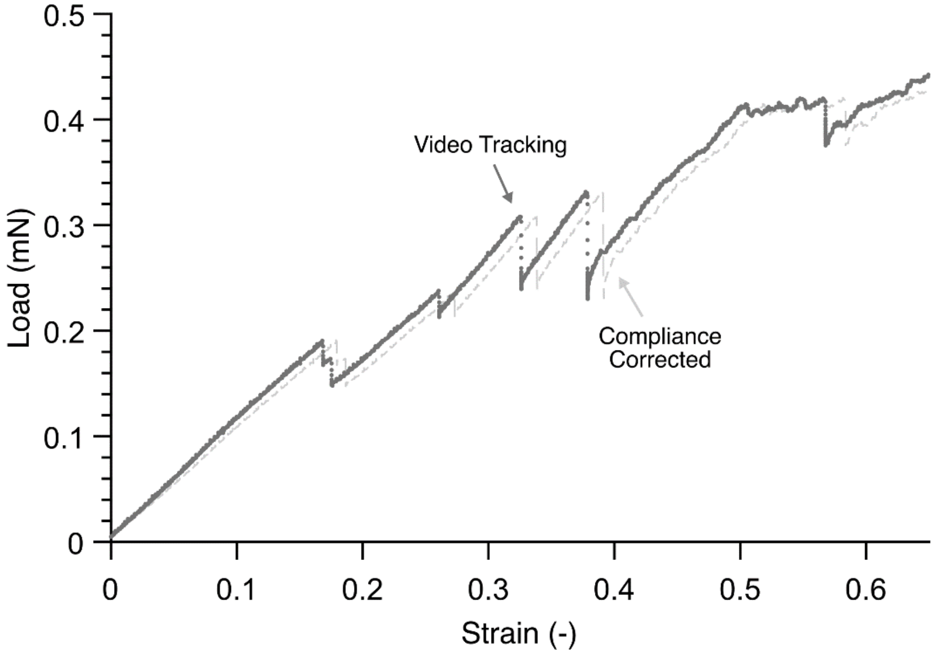

Fig. S6.

Comparison between data correction methods. Load vs. strain data from a rhombus of *H* = 70 μm tested in tension up to failure and corrected using (i) the compliance correction method and (ii) digital image tracking. The lack of deviation between the two data sets shows that both methods are comparable to one another. Note that charging on polymer surface during prolonged imaging inside an SEM may induce measurement errors when using digital image tracking.

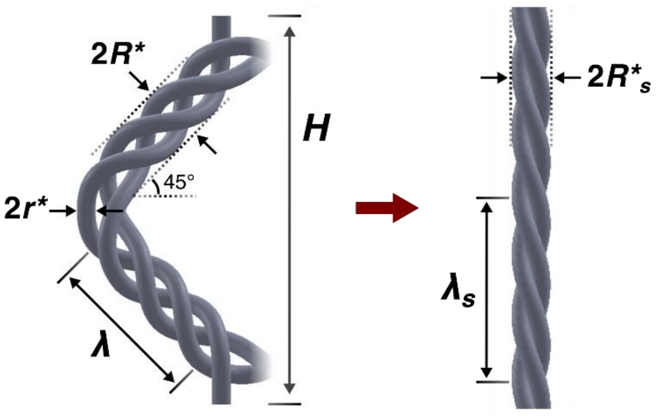

Fig. S7.

Geometrical parameters in fiber straightening. Computer-aided design (CAD) models of undeformed (left) and straightened (right) strands of fibers with relevant geometrical parameters.

Table S1.

XPS characterization of pristine IP-Dip. Assignments to a C 1s signal from XPS characterization of a pristine IP-Dip plate shown in Fig. S4, A.

Assignment	Line Shape	Intensity (RSF Adj.)	FWHM (eV)	FWHM Const. (eV)	Position (eV)	Area %
C-C, C-H	LA	2348.4	1.4	0.8-1.4	282.2	45.83
C-O	LA	1332.1	1.4	0.8-1.4	283.5	25.95
C=O	LA	364.9	1.3	0.8-1.4	284.9	7.14
O-C=O	LA	944.3	1.1	0.8-1.4	286.1	18.41
π-π*	LA	137.2	3.4	-	288.1	2.68

Table S2.

XPS characterization of 29-hr UV-irradiated IP-Dip. Assignments to a C 1s signal from XPS characterization of a 29-hr UV-irradiated IP-Dip plate shown in Fig. S4, B.

Assignment	Line Shape	Intensity (RSF Adj.)	FWHM (eV)	FWHM Const. (eV)	Position (eV)	Area %
C-C, C-H	LA	3539.5	1.4	0.8-1.4	282.2	47.14
C-O	LA	1927.8	1.4	0.8-1.4	283.6	25.67
C=O	LA	546.6	1.2	0.8-1.4	284.8	7.28
O-C=O	LA	1402.6	1.2	0.8-1.4	286.2	18.68
π-π*	LA	92.3	1.7	-	288.6	1.23

Table S3.

XPS characterization of pristine IP-Dip from a separate batch. Assignments to a C 1s signal from XPS characterization of a pristine IP-Dip plate shown in Fig. S4, C.

Assignment	Line Shape	Intensity (RSF Adj.)	FWHM (eV)	FWHM Const. (eV)	Position (eV)	Area %
C-C, C-H	LA	4483.4	1.4	0.8-1.4	282.2	47.11
C-O	LA	2697.8	1.4	0.8-1.4	283.4	25.13
C=O	LA	1108.4	1.1	0.8-1.4	284.1	10.33
O-C=O	LA	2113.3	1.2	0.8-1.4	286.3	19.69
π-π*	LA	330.9	5.3	-	286.9	3.083

Table S4.

XPS characterization of 29-hr UV-irradiated IP-Dip from a separate batch. Assignments to a C 1s signal from XPS characterization of a 29-hr UV-irradiated IP-Dip plate shown in Fig. S4, D.

Assignment	Line Shape	Intensity (RSF Adj.)	FWHM (eV)	FWHM Const. (eV)	Position (eV)	Area %
C-C, C-H	LA	4623.1	1.3	0.8-1.4	282.6	44.02
C-O	LA	3300.2	1.3	0.8-1.4	284.0	31.42
C=O	LA	187.1	0.84	0.8-1.4	285.3	1.78
O-C=O	LA	2392.5	1.1	0.8-1.4	286.5	22.78
π-π*	LA	1.9×10^{-20}	7.58	-	291.4	0.0

Movie S1.
Video of hierarchical knotted (left, purple) and woven (right, red) rhombuses with designed height H = 70 μm pulled up to failure played at 75x speed, showing a unique knotting mechanism only available in hierarchical knotted rhombus once fibers are aligned to the loading direction.

Movie S2.
Video of hierarchical knotted (left, purple) and woven (right, red) rhombuses with designed height H = 70 μm cyclically loaded in tension to an increasing strain value in each subsequent cycle with 300x playback speed. Within the first two cycles, both rhombuses returned to shapes close to their undeformed configurations. In the third cycle, the woven rhombus experienced failure whereas the knotted rhombus was tightened and retained its shape after unloading.

Movie S3.
Video of hierarchical knotted rhombuses of height H = 70 μm (left, dark purple) and 140 μm (right, light purple) loaded in tension up to failure played at 100x speed. The normalized load ($\bar{F}$) is defined as the applied load divided by the product of the Young's Modulus of pristine IP-Dip and the fiber cross-sectional area.

REFERENCES

1. U. G. K. Wegst, H. Bai, E. Saiz, A. P. Tomsia, R. O. Ritchie, Bioinspired structural materials. *Nat. Mater.* **14**, 23–36 (2015).

2. O. A. Tertuliano, B. W. Edwards, L. R. Meza, V. S. Deshpande, J. R. Greer, Nanofibril-mediated fracture resistance of bone. *Bioinspir. Biomim.* **16**, 035001 (2021).

3. M. E. Launey, M. J. Buehler, R. O. Ritchie, On the mechanistic origins of toughness in bone. *Annu. Rev. Mat. Res.* **40**, 25–53 (2010).

4. A. Miserez, J. C. Weaver, P. J. Thurner, J. Aizenberg, Y. Dauphin, P. Fratzl, D. E. Morse, F. W. Zok, Effects of laminate architecture on fracture resistance of sponge biosilica: Lessons from nature. *Adv. Funct. Mater.* **18**, 1241–1248 (2008).

5. T. A. Schaedler, A. J. Jacobsen, A. Torrents, A. E. Sorensen, J. Lian, J. R. Greer, L. Valdevit, W. B. Carter, Ultralight metallic microlattices. *Science* **334**, 962–965 (2011).

6. L. R. Meza, S. Das, J. R. Greer, Strong, lightweight, and recoverable three-dimensional ceramic nanolattices. *Science* **345**, 1322–1326 (2014).

7. X. Zheng, W. Smith, J. Jackson, B. Moran, H. Cui, D. Chen, J. Ye, N. Fang, N. Rodriguez, T. Weisgraber, C. M. Spadaccini, Multiscale metallic metamaterials. *Nat. Mater.* **15**, 1100–1106 (2016).

8. S. Shi, Y. Li, B. N. Ngo-Dinh, J. Markmann, J. Weissmüller, Scaling behavior of stiffness and strength of hierarchical network nanomaterials. *Science* **371**, 1026–1033 (2021).

9. S. Krödel, C. Daraio, Microlattice metamaterials for tailoring ultrasonic transmission with elastoacoustic hybridization. *Phys. Rev. Appl.* **6**, 064005 (2016).

10. A. Frölich, J. Fischer, T. Zebrowski, K. Busch, M. Wegener, Titania woodpiles with complete three-dimensional photonic bandgaps in the visible. *Adv. Mater.* **25**, 3588–3592 (2013).

11. Q. Wang, J. A. Jackson, Q. Ge, J. B. Hopkins, C. M. Spadaccini, N. X. Fang, Lightweight mechanical metamaterials with tunable negative thermal expansion. *Phys. Rev. Lett.* **117**, 175901 (2016).

12. J. A. Harris, R. E. Winter, G. J. McShane, Impact response of additively manufactured metallic hybrid lattice materials. *Int. J. Impact Eng.* **104**, 177–191 (2017).

13. C. M. Portela, B. W. Edwards, D. Veysset, Y. Sun, K. A. Nelson, D. M. Kochmann, J. R. Greer, Supersonic impact resilience of nanoarchitected carbon. *Nat. Mater.* **20**, 1491–1497 (2021).

14. R. Lakes, Materials with structural hierarchy. *Nature* **361**, 511–515 (1993).

15. L. R. Meza, A. J. Zelhofer, N. Clarke, A. J. Mateos, D. M. Kochmann, J. R. Greer, Resilient 3D hierarchical architected metamaterials. *Proc. Natl. Acad. Sci. U.S.A.* **112**, 11502–11507 (2015).

16. D. Rayneau-Kirkhope, Y. Mao, R. Farr, Ultralight fractal structures from hollow tubes. *Phys. Rev. Lett.* **109**, 204301 (2012).

17. A. Ajdari, B. H. Jahromi, J. Papadopoulos, H. Nayeb-Hashemi, A. Vaziri, Hierarchical honeycombs with tailorable properties. *Int. J. Solids Struct.* **49**, 1413–1419 (2012).

18. R. Oftadeh, B. Haghpanah, D. Vella, A. Boudaoud, A. Vaziri, Optimal fractal-like hierarchical honeycombs. *Phys. Rev. Lett.* **113**, 104301 (2014).

19. G. W. Kooistra, V. Deshpande, H. N. G. Wadley, Hierarchical corrugated core sandwich panel concepts. *J. Appl. Mech. Trans.* **74**, 259–268 (2007).

20. S. Malek, L. Gibson, Effective elastic properties of periodic hexagonal honeycombs. *Mech. Mater.* **91**, 226–240 (2015).

21. M. T. Hsieh, B. Endo, Y. Zhang, J. Bauer, L. Valdevit, The mechanical response of cellular materials with spinodal topologies. *J. Mech. Phys. Solids* **125**, 401–419 (2019).

22. C. M. Portela, A. Vidyasagar, S. Krödel, T. Weissenbach, D. W. Yee, J. R. Greer, D. M. Kochmann, Extreme mechanical resilience of self-assembled nanolabyrinthine materials. *Proc. Natl. Acad. Sci. U.S.A.* **117**, 5686–5693 (2020).

23. L. R. Meza, G. P. Phlipot, C. M. Portela, A. Maggi, L. C. Montemayor, A. Comella, D. M. Kochmann, J. R. Greer, Reexamining the mechanical property space of three-dimensional lattice architectures. *Acta Mater.* **140**, 424–432 (2017).

24. J. B. Berger, H. N. G. Wadley, R. M. McMeeking, Mechanical metamaterials at the theoretical limit of isotropic elastic stiffness. *Nature* **543**, 533–537 (2017).

25. C. Crook, J. Bauer, A. Guell Izard, C. Santos de Oliveira, J. Martins de Souza e Silva, J. B. Berger, L. Valdevit, Plate-nanolattices at the theoretical limit of stiffness and strength. *Nat. Commun.* **11**, 1579 (2020).

26. T. Tancogne-Dejean, D. Mohr, Elastically-isotropic elementary cubic lattices composed of tailored hollow beams. *Extreme Mech. Lett.* **22**, 13–18 (2018).

27. S. C. Han, J. W. Lee, K. Kang, A new type of low density material: Shellular. *Adv. Mater.* **27**, 5506–5511 (2015).

28. C. Bonatti, D. Mohr, Mechanical performance of additively-manufactured anisotropic and isotropic smooth shell-lattice materials: Simulations & experiments. *J. Mech. Phys. Solids* **122**, 1–26 (2019).

29. S. Shan, S. H. Kang, J. R. Raney, P. Wang, L. Fang, F. Candido, J. A. Lewis, K. Bertoldi, Multistable architected materials for trapping elastic strain energy. *Adv. Mater.* **27**, 4296–4301 (2015).

30. B. Haghpanah, L. Salari-Sharif, P. Pourrajab, J. Hopkins, L. Valdevit, Multistable shape-reconfigurable architected materials. *Adv. Mater.* **28**, 7915–7920 (2016).

31. N. A. Traugutt, D. Mistry, C. Luo, K. Yu, Q. Ge, C. M. Yakacki, Liquid-crystal-elastomer-based dissipative structures by digital light processing 3D printing. *Adv. Mater.* **32**, 2000797 (2020).

32. T. Frenzel, C. Findeisen, M. Kadic, P. Gumbsch, M. T. Wegener Frenzel, M. Kadic, M. Wegener, C. Findeisen, P. Gumbsch, Tailored buckling microlattices as reusable light-weight shock absorbers. *Adv. Mater.* **28**, 5865–5870 (2016).

33. L. Salari-Sharif, L. Valdevit, T. A. Schaedler, Energy dissipation mechanisms in hollow metallic microlattices. *J. Mater. Res.* **29**, 1755–1770 (2014).

34. B. C. White, A. Garland, R. Alberdi, B. L. Boyce, Interpenetrating lattices with enhanced mechanical functionality. *Addit. Manuf.* **38**, 101741 (2021).

35. P. P. Indurkar, A. Shaikeea, Z. Xu, H. Cui, X. Zheng, V. Deshpande, The coupled strength and toughness of interconnected and interpenetrating multi-material gyroids. *MRS Bull.* **47**, 461–473 (2022).

36. S. M. Ryan, S. Szyniszewski, S. Ha, R. Xiao, T. D. Nguyen, K. W. Sharp, T. P. Weihs, J. K. Guest, K. J. Hemker, Damping behavior of 3D woven metallic lattice materials. *Scr. Mater.* **106**, 1–4 (2015).

37. L. Salari-Sharif, S. M. Ryan, M. Pelacci, J. K. Guest, L. Valdevit, S. Szyniszewski, Damping of selectively bonded 3D woven lattice materials. *Sci. Rep.* **8**, 14572 (2018).

38. A. P. Garland, K. M. Adstedt, Z. J. Casias, B. C. White, W. M. Mook, B. Kaehr, B. H. Jared, B. T. Lester, N. S. Leathe, E. Schwaller, B. L. Boyce, Coulombic friction in metamaterials to dissipate mechanical energy. *Extreme Mech. Lett.* **40**, 100847 (2020).

39. J. Li, Z. Chen, Q. Li, L. Jin, Z. Zhao, Harnessing friction in intertwined structures for high-capacity reusable energy-absorbing architected materials. *Adv. Sci.* **9**, 2105769 (2022).

40. W. P. Moestopo, A. J. Mateos, R. M. Fuller, J. R. Greer, C. M. Portela, Pushing and pulling on ropes: Hierarchical woven materials. *Adv. Sci.* **7**, 2001271 (2020).

41. S. Pellegrino, Structural computations with the singular value decomposition of the equilibrium matrix. *Int. J. Solids Struct.* **30**, 3025–3035 (1993).

42. Y. Li, S. Pellegrino, A theory for the design of multi-stable morphing structures. *J. Mech. Phys. Solids* **136**, 103772 (2020).

43. V. V. Rybenkov, N. R. Cozzarelli, A. V. Vologodskii, Probability of DNA knotting and the effective diameter of the DNA double helix. *Proc. Natl. Acad. Sci. U.S.A.* **90**, 5307–5311 (1993).

44. Y. Arai, R. Yasuda, K. I. Akashi, Y. Harada, H. Miyata, K. Kinosita, H. Itoh, Tying a molecular knot with optical tweezers. *Nature* **399**, 446–448 (1999).

45. M. D. Frank-Kamenetskii, A. V. Lukashin, A. V. Vologodskii, Statistical mechanics and topology of polymer chains. *Nature* **258**, 398–402 (1975).

46. A. M. Saitta, P. D. Soper, E. Wasserman, M. L. Klein, Influence of a knot on the strength of a polymer strand. *Nature* **399**, 46–48 (1999).

47. R. H. Crowell, R. H. Fox, *Introduction to Knot Theory* (Springer, 1963).

48. B. Polster, Mathematics: What is the best way to lace your shoes? *Nature* **420**, 476 (2002).

49. C. A. Daily-Diamond, C. E. Gregg, O. M. O'Reilly, The roles of impact and inertia in the failure of a shoelace knot. *Proc. Math. Phys. Eng. Sci.* **473**, 20160770 (2017).

50. V. P. Patil, J. D. Sandt, M. Kolle, J. Dunkel, Topological mechanics of knots and tangles. *Science* **367**, 71–75 (2020).

51. H. Uehara, H. Kimura, A. Aoyama, T. Yamanobe, T. Komoto, Effects of knot characteristics on tensile breaking of a polymeric monofilament. *New J. Phys.* **9**, 65 (2007).

52. S. Przybyl, P. Pieranski, Tightening of the elastic overhand knot. *Phys. Rev. E* **79**, 031801 (2009).

53. P. Johanns, P. Grandgeorge, C. Baek, T. G. Sano, J. H. Maddocks, P. M. Reis, The shapes of physical trefoil knots. *Extreme Mech. Lett.* **43**, 101172 (2021).

54. M. K. Jawed, P. Dieleman, B. Audoly, P. M. Reis, Untangling the mechanics and topology in the frictional response of long overhand elastic knots. *Phys. Rev. Lett.* **115**, 118302 (2015).

55. N. M. Pugno, The "Egg of Columbus" for making the world's toughest fibres. *PLOS ONE* **9**, e93079 (2014).

56. A. Berardo, M. F. Pantano, N. M. Pugno, Slip knots and unfastening topologies enhance toughness without reducing strength of silk fibroin fibres. *Interface Focus* **6**, 20150060 (2016).

57. M. F. Pantano, A. Berardo, N. M. Pugno, Tightening slip knots in raw and degummed silk to increase toughness without losing strength. *Sci. Rep.* **6**, 1–8 (2016).

58. N. Maeda, N. Chen, M. Tirrell, J. N. Israelachvili, Adhesion and friction mechanisms of polymer-on-polymer surfaces. *Science* **297**, 379–382 (2002).

59. J. Bauer, A. Guell Izard, Y. Zhang, T. Baldacchini, L. Valdevit, J. Bauer, A. Guell Izard, L. Valdevit, Y. Zhang, T. Baldacchini, Programmable mechanical properties of two-photon polymerized materials: From nanowires to Bulk. *Adv. Mater. Technol.* **4**, 1900146 (2019).

60. J. Bauer, A. Schroer, R. Schwaiger, O. Kraft, The impact of size and loading direction on the strength of architected lattice materials. *Adv. Eng. Mater.* **18**, 1537–1543 (2016).

61. C. D. McClure, C. J. Oldham, G. N. Parsons, Effect of Al_2O_3 ALD coating and vapor infusion on the bulk mechanical response of elastic and viscoelastic polymers. *Surf. Coat. Technol.* **261**, 411–417 (2015).

62. J. Bauer, A. G. Izard, Y. Zhang, T. Baldacchini, T. Baldacchini, L. Valdevit, Thermal post-curing as an efficient strategy to eliminate process parameter sensitivity in the mechanical properties of two-photon polymerized materials. *Opt. Express* **28**, 20362–20371 (2020).

63. L. C. Montemayor, W. H. Wong, Y. W. Zhang, J. R. Greer, Insensitivity to flaws leads to damage tolerance in brittle architected meta-materials. *Sci. Rep.* **6**, 20570 (2016).

64. X. Zhang, A. Vyatskikh, H. Gao, J. R. Greer, X. Li, Lightweight, flaw-tolerant, and ultrastrong nanoarchitected carbon. *Proc. Natl. Acad. Sci. U.S.A.* **116**, 6665–6672 (2019).

65. T. R. Gengenbach, G. H. Major, M. R. Linford, C. D. Easton, Practical guides for x-ray photoelectron spectroscopy (XPS): Interpreting the carbon 1s spectrum. *J. Vac. Sci. Technol. A* **39**, 013204 (2021).

66. A. G. Shard, Practical guides for x-ray photoelectron spectroscopy: Quantitative XPS. *J. Vac. Sci. Technol A* **38**, 041201 (2020).
